# Profiling Physical Activity, Diet, Screen and Sleep Habits in Portuguese Children

**DOI:** 10.3390/nu7064345

**Published:** 2015-06-02

**Authors:** Sara Pereira, Peter T. Katzmarzyk, Thayse Natacha Gomes, Alessandra Borges, Daniel Santos, Michele Souza, Fernanda K. dos Santos, Raquel N. Chaves, Catherine M. Champagne, Tiago V. Barreira, José A.R. Maia

**Affiliations:** 1 CIFI^2^D, Faculty of Sport, University of Porto, Rua Plácido Costa, 91, 4200-450 Porto, Portugal; E-Mails: sara.s.p@hotmail.com (S.P.); thayse_natacha@hotmail.com (T.N.G.); borges_alessandra@hotmail.com (A.B.); d.monteiro.santos13@gmail.com (D.S.); mcsouza85@hotmail.com (M.S.); 2 Pennington Biomedical Research Center, Louisiana State University System, Baton Rouge, LA 70808, USA; E-Mails: peter.katzmarzyk@pbrc.edu (P.T.K.); catherine.champagne@pbrc.edu (C.M.C.); tiago.barreira@pbrc.edu (T.V.B.); 3 Department of Physical Education and Sports Science, CAV, Federal University of Pernambuco, Vitória de Santo Antão 55608-680, Brazil; E-Mail: fernandak.santos@hotmail.com; 4 Federal University of Technology—Paraná (UTFPR), Campus Curitiba, Curitiba 80230-901, Brazil; E-Mail: raquelnichele@live.com.pt; 5 School of Education, Syracuse University, Syracuse, NY 13244, USA

**Keywords:** unhealthy lifestyle behaviours, latent classes, youth, ISCOLE

## Abstract

Obesity in children is partly due to unhealthy lifestyle behaviours, e.g., sedentary activity and poor dietary choices. This trend has been seen globally. To determine the extent of these behaviours in a Portuguese population of children, 686 children 9.5 to 10.5 years of age were studied. Our aims were to: (1) describe profiles of children’s lifestyle behaviours; (2) identify behaviour pattern classes; and (3) estimate combined effects of individual/socio-demographic characteristics in predicting class membership. Physical activity and sleep time were estimated by 24-h accelerometry. Nutritional habits, screen time and socio-demographics were obtained. Latent Class Analysis was used to determine unhealthy lifestyle behaviours. Logistic regression analysis predicted class membership. About 78% of children had three or more unhealthy lifestyle behaviours, while 0.2% presented no risk. Two classes were identified: Class 1-Sedentary, poorer diet quality; and Class 2-Insufficiently active, better diet quality, 35% and 65% of the population, respectively. More mature children (Odds Ratio (OR) = 6.75; 95%CI = 4.74–10.41), and boys (OR = 3.06; 95% CI = 1.98–4.72) were more likely to be overweight/obese. However, those belonging to Class 2 were less likely to be overweight/obese (OR = 0.60; 95% CI = 0.43–0.84). Maternal education level and household income did not significantly predict weight status (*p* ≥ 0.05).

## 1. Introduction

Unhealthy behaviours such as low physical activity (PA) levels, high screen time, poor diet, and short sleep duration have been associated with cardiovascular disease risk factors, obesity, and other poor health outcomes in children [1,2,3]. These associations have mostly been studied in bivariate terms [1,2]. For example, Carson *et al.* (2014) found no association between sedentary time and BMI *z*-score in Canadian children, whereas moderate-to-vigorous PA (MVPA) was consistently associated with BMI *z*-score [1]. Further, Kell *et al.* (2014) concluded that increased consumption of added sugars may be associated with adverse cardiovascular health factors, especially elevated diastolic blood pressure and triglycerides in a multi-ethnic pediatric sample aged 7–12 years [2]. Additionally, sleep is a behaviour that has been linked to several health outcomes in children. Particularly relevant are the associations between short sleep duration, obesity [4] and cardiometabolic risk [5,6] in children.

In Portugal, information concerning nutrition and health behaviours among children is somewhat scarce. For example, Stamatakis *et al.* (2013), studying the relationship between screen time and adiposity in Portuguese children aged 2–13 years old, found a positive association between watching TV and body fat [7]. On the other hand, Valente *et al.* (2009) investigated the association between sugar-sweetened beverage (SSB) consumption with weight status in Portuguese children aged 5–10 years and found no significant difference in energy intake between overweight and normal weight children. However, when considering the percentage contributions to total energy intake, no differences were found between normal weight and overweight girls, but significantly higher mean intakes of total carbohydrates and sugars were found in overweight boys compared with normal weight ones [8]. In addition, in a recent meta-analysis involving studies with Portuguese children published between 2000 and 2014, Gomes *et al.* (2014) reported that the prevalence of overweight/obesity ranged, in this period, between 19% and 35%, meaning moderate to high values [9].

Studying not only interactions among unhealthy behaviours and health risks, but also identifying behavioral configurations using the concept of clustering or group risk profiling/patterning has been suggested over recent years [3]. For example, Fernández-Alvira *et al.* (2013) used data from seven European countries and identified five distinct clustered groups based on sugary drink consumption, PA levels, screen time, and sleep duration [10]. The cluster with high physical activity level showed the highest proportion of participants with highly educated parents, while clusters with high sugary drink consumption, high screen time and low sleep duration were more prevalent in the group with less educated parents. Similarly, a study by Jago *et al.* (2010) identified children’s risk behavior clusters (PA and sedentary time), and found that three groups emerged from their analysis. The most prevalent cluster “Low Active/ Medium Sedentary group” (45.5%) was characterized by low levels of physical activity and moderate time in sedentary behaviours [11].

Patterns/profiles of healthy behaviours (based on sleep duration, PA, screen time and diet) were also studied in Australian children by Magee *et al.* (2013), who identified three behavioral profiles: the first (27.7%) was termed “healthy” because they consumed more fruits and vegetables, the lowest level of fat food consumption and low levels of high sugary drink consumption; the second profile (24.8%) was labelled “sedentary” because higher rates of physical inactivity and screen time were found; the third profile (47.5%) was labelled “short sleepers/unhealthy eaters” because children tended to consume high fat, sugary foods and drinks and had the highest percentage of children with <10 h/night of sleep time [12]. When comparing the healthy profile with the other two (sedentary profile and short sleepers/unhealthy eaters), the latter two groups had elevated odds for becoming obese at two years of follow-up [12]. These examples show the importance of considering risk factor clustering in terms of public health, education, and prevention because it will foster a better understanding about which set of joint behaviours needs to be simultaneously changed in order to improve children’s health. However, in a recent review, Leech *et al.* (2014) reported some inconsistency in relation to the effect of these clustered behavior patterns on obesity since some studies found higher prevalence of overweight/obesity in unhealthy clusters, whereas others found no association at all. Further research in this area is clearly needed. In the present study, our aim was to identify and describe the clustering of lifestyle behaviours using latent class analysis and estimate the effects of both individual and socio-demographic characteristics in predicting class membership [3].

## 2. Experimental Section

### 2.1. Material and Methods

Data for the present paper were collected during the International Study of Childhood Obesity, Lifestyle and the Environment (ISCOLE), a research project conducted at sites in 12 countries from all major world regions. Details of the overall study design have been previously reported by Katzmarzyk *et al.* (2013) [13]. Briefly, the sample for the present study includes 686 Portuguese 5th grade children (381 girls and 305 boys), aged 9–11 years, who were randomly selected from 23 schools (*ca.* 30–40 students/per school) in the Oporto metropolitan area, in northern Portugal. Non-response was negligible (response rate was 95.7%); since missing information was at random we did not use any imputation (single or multiple) to fill-in the missing values. The primary sampling unit was schools and the secondary sampling unit was classes in the school that best corresponded to 10-year-old students. Schools were randomly selected from a list provided by the North Regional Education Directory Board, belonging to different neighborhoods from Porto, with the purpose of including schools from different neighborhood socioeconomic status. If a school was non-compliant school, it was replaced by the next random school selected from the group. Since ISCOLE included only children aged 9–11 years, the 5th grade students were selected.

Detailed information about the study was sent to all parents, and written consent was obtained from the parents as well as assent to participate from the children. The Oporto University Ethics Committee approved the project. Further, all data were collected by certified personnel trained by the ISCOLE coordinating center under highly controlled conditions as reported elsewhere [13].

#### 2.1.1. Anthropometry

Height and sitting height (cm) were measured using a Seca 213 portable stadiometer (Hamburg, Germany), with the head positioned in the Frankfurt plane. Weight (kg), and percentage of body fat were measured with children in light clothing, using a portable Tanita SC-240 body composition analyzer (Hellington Heights, USA). Waist circumference (cm) was measured at the end of gentle expiration with a non-elastic tape held midway between the lower rib margin and the iliac crest. The anthropometry measurement protocol was previously described [13]. Body mass index (BMI) was computed using the standard formula (body mass (kg)/height (m)^2^), and children were categorized as normal weight and overweight/obese according to cut points defined by the WHO [14]. All measurements were made by trained researchers under standardized conditions [13].

#### 2.1.2. Maturity Offset

Biological maturation was indirectly estimated with the maturity offset procedure proposed by Mirwald *et al.* (2002) using sex-specific regression equations with individual data from height, weight and sitting height [15]. The maturity offset estimates the temporal distance that each subject is from peak height velocity (PHV) using chronological age with the value expressed in decimal years. A positive (+) maturity offset represents the number of years the participant is beyond PHV, whereas a negative (–) maturity offset represents the number of years the subject is prior to PHV.

#### 2.1.3. Physical Activity and Sleep Time

PA and sleep time were objectively assessed with the Actigraph GT3X+ accelerometer (ActiGraph LLC, Pensacola, FL, USA), for 24 h/day on 7 consecutive days (including 2 weekend days), and only removed during water activities (*i.e.*, showering, swimming). The accelerometer was attached to the participant using an elastic belt worn around the waist with an adjustable clip. The accelerometer unit was placed in line with the mid-axillary line and lying on the iliac crest (*i.e.*, hip location).). ActiLife software was used to download recorded data immediately upon retrieval of each accelerometer. The downloading process produced an AgileGraph Data File (AGD file) with the following settings: 1 s epoch, 3 axes of orientation, steps, lux (ambient light), inclinometer, and low frequency extension (LFE) [13]. The total average wear time in the sample was 22.7 h per day. After removal of sleep time, average “awake” wear time was 15.2 h per day. The minimal amount of accelerometer data that was considered acceptable was 4 days with at least 10 h of awake wear time per day, including at least one weekend day; all 686 children completed this requirement.

PA variables were derived using activity counts as advocated by Evenson *et al.* (2008). For this study, only the average weekly time (expressed in minutes) spent in MVPA was used, and defined as greater than 574 activity counts per 15 s [16]. Children were categorized into two groups (<60 min·day^−1^; ≥60 min·day^−1^) based on their compliance with MVPA daily recommendations [17].

Sleep time was estimated as proposed by Tudor-Locke *et al.* (2014) and refined by Barreira *et al.* (2014) [18,19]. Sleep time was estimated across all valid days (expressed in hours) and children were categorized into two groups (<600 min·night^−1^; ≥600 min·night^−1^), according to the daily recommendation for sleep time proposed by the National Sleep Foundation (2013) [20].

Accelerometer data were first divided into awake time and nocturnal sleep time using an automated algorithm [15,16]. After exclusion of the nocturnal sleep period, walking non-wear time was defined as any sequence of at least 20 consecutive minutes of zero activity counts [19].

#### 2.1.4. Fruit, Vegetable and Sugary Drink Consumption

The information related to fruit, vegetable and sugary drink consumption was obtained from a food frequency questionnaire (FFQ) [13]. Dietary intake patterns were assessed using a food frequency questionnaire (FFQ) adapted from the Health Behavior in School-aged Children Survey (HBSC) [21] and adapted by inclusion of a variety of typical Portuguese food items. Children were asked about several different types of food consumed in a usual week. The FFQ lists 23 food categories and has examples of individual food items, but without portion sizes. The answer options were: never; less than once per week; once per week; 2–4 days per week; 5–6 days per week; once a day every day; and more than once a day. For fruit/vegetable consumption, children’s responses were divided into two groups—those consuming this type of food every day of the week and those who did not (the former was considered the healthy lifestyle behaviour group); as for sugary drink consumption, children were also divided into two groups—those who consumed less than two times per week *versus* those who consumed two or more times (the latter was considered the unhealthy lifestyle behaviour group). This information was administered by ISCOLE Staff and completed by the children at school. Questionnaires were checked for completeness at the time of data collection in order to ensure high quality in data acquisition.

#### 2.1.5. Screen Time

Information regarding screen time was derived from time (in hours) spent during weekdays in (1) TV watching and (2) playing non-active video games or using the computer, which were obtained from a questionnaire for which the responses were categories (not watching TV or playing video games or using the computer/<1 h/1 h/2 h/3 h/4 h/5 h or more) [13]. Answers provided by children for both questions were summed and then categorized according to screen time recommendations for children (<120 min·day^−1^; ≥ 120 min·day^−1^) [22].

#### 2.1.6. Socio-Demographic Characteristics

A demographic and family health history questionnaire containing information about the household, such as maternal education and household income [13], was completed by parents, self-administered at home. Maternal education and household income were used as proxies for socioeconomic status (SES) as follows: maternal education level (category1: <Grade 12; category 2: Grade 12/diploma for technical qualification (equivalent to high school); category 3: University level); for annual household income (category 1: <12.000 €; category 2: between 12.000 € and 29.999 €; category 3: ≥30.000 €).

### 2.2. Statistical Analysis

Basic statistics were computed in SPSS 20. All five behaviours were coded as 0 (risk not present: MVPA ≥ 60 min·day^−1^, screen time < 120 min·day^−1^, fruit and vegetable consumption all days, sleep ≥ 600 min·night^−1^, sugary drinks < two days·week^−1^) or 1 (risk present). To describe unhealthy lifestyle behaviour patterns, an exploratory Configural Frequency Analysis (CFA) was performed as implemented in CFA software. A base model called Configural Cluster Analysis, which assumes that all configurations (25 = 32 possible configurations) have the same probability, *i.e.*, the same expected frequency, was used as previously advocated [23].

Using Mplus v. 6 iterative maximum likelihood estimation techniques, a Latent Class Analysis was performed to identify the number of unobserved subgroups comprising individuals of similar behavioral risk. Latent Class Analysis focusing on grouping respondents, or case based on patterns of item (behaviours) responses’ is considered a person-centered approach [24]. As previously advocated [24,25], model fitting assessment and model comparisons utilized the Pearson χ^2^ statistic, as a measure of absolute fit, and the bootstrap likelihood ratio difference test, the Lo-Mendell-Rubin adjusted test (LRT), the Akaike Information Criteria (AIC), and the Bayesian Information Criterion (BIC) as measures of relative fit when comparing different numbers of latent class models. Model comparison was established as follows: firstly a most parsimonious model with only 1 class was fitted; then successive models with an increase in the number of classes, up to 4 were estimated. To avoid identification problems related to local maxima, the algorithm iterations were set as suggested by Geiser (2013), and Wang and Wang (2012) [24,25]. The best fitting model is the one with lower values of relative fit measures, and has a substantive interpretation. Using the new classification classes, we then used logistic regression to predict weight status having sex (0 = girls; 1 = boys), BMI, maturity offset, mother’s education and household income as predictors and class membership, after checking for multicollinearity. This analysis accounted for by schools clustering effects. Stata 13 software was used.

## 3. Results

Table 1 displays descriptive statistics regarding the sample population. On average, children were 143.5 cm tall, weighed 40.4 kg, had 22.9% body fat, a BMI of 19.5 kg/m^2^ and were 1.9 years away from age at PHV. Further, 15.7% of children’s mothers have a University degree, and 39.4% of children’s families have a low household income (less than 12,000 € per year).

When considering individual unhealthy lifestyle behaviours, 63.6% of the children did not meet the PA recommendation 1 of daily MVPA (60 min·day^−1^). Moreover, 63.4% spent more than 120 min·day^−1^ on weekdays in screen time, 71.3% did not consume fruits/vegetables on all days, 35.3% consumed sugary drinks more than two days·week^−1^, and 92.7% had less than 600 min of sleep·night^−1^.

Table 2 contains descriptive information regarding the Configural Cluster Analysis base model with its 32 possible configurations. There is a wide variation in configurations when the joint risk factors are considered, from only 1 case (≈0.2%) with no unhealthy lifestyle behaviours to 67 cases (≈10%) with all five unhealthy lifestyle behaviours. Additionally, the frequency of three or more unhealthy lifestyle behaviours was found in 533 cases (78%).

**Figure 1:**
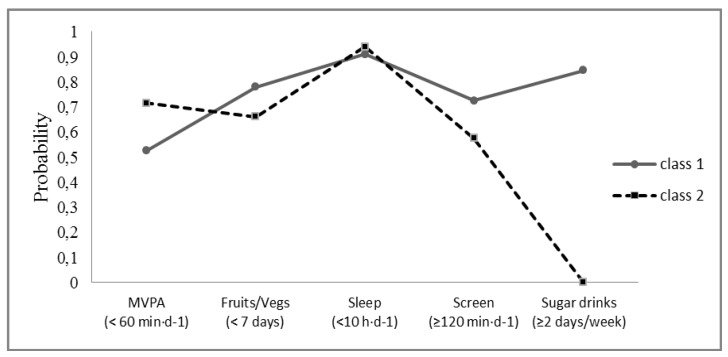
Profiles for the 2-class Latent class analysis (LCA) model of risk behaviours.

Information regarding the test criteria to find the best number of latent classes can be found in Table 3. The most parsimonious model with only a single class was rejected, favoring a two class model. Thus, there was no statistical justification to go beyond a 2-latent class model based on the best fit measures (lower relative fit and more parsimonious model given also the number of free parameters). Figure 1 illustrates the maximum likelihood solution to display the conditional probabilities of the 2-class model (for details, see [22]). Given the probabilities of behavioral risk, we labelled class 1 as “sedentary, poorer diet quality”; class 2 was labelled as “insufficiently active, better diet quality”.

**Table 1:** Sample descriptive characteristics.

Variables	Total
	*n* (%) or mean ± sd
Anthropometric sample characteristics	
Height (cm)	143.5 ± 6.8
Weight (kg)	40.4 ± 9.2
Percent body fat (%)	22.9 ± 7.5
BMI (Kg/m^−2^) need to footnote this	19.5 ± 3.4
Maturity offset	−1.90 ± 0.9
Gender	
Boys	305 (44.5%)
Girls	381 (55.5%)
Weight Status	
Normal weight	372 (54.2%)
Overweight/obese	314 (45.8%)
Maternal education	
<Grade 12	317 (46.2%)
Grade 12/diploma/technical qualification	191 (27.8%)
University	108 (15.7%)
Did not report	70 (10.2%)
Household income	
<12.000 €	270 (39.4%)
12.000 €–29.999 €	195 (28.4%)
≥30.000 €	76 (11.1%)
Did not report	145 (21.1%)

Frequencies of behaviours, biological and demographic characteristics of the two latent classes, as well as their individual differences based on a chi-square test are found in Table 4. Class 1 was significantly (*p* ≤ 0.05) less inactive, slept more, drank more sugary drinks, ate less fruits/vegetables and had higher screen time than Class 2. The proportion of boys and girls was significantly different (*p* = 0.004) between the groups, with a greater proportion of girls being classified in Class 2, while a greater proportion of boys were categorized in Class 1; but no between group significant differences were observed regarding weight status. Significant differences were observed in maternal education, as a higher prevalence of mothers with a University degree was found in Class 2; however, no significant difference was found in household income categories between the two classes.

Results of the logistic regression analysis are presented in Table 5. Boys were more likely to be overweight/obese than girls (odds ratio (OR) = 3.06, 95% Confidence Interval (CI) = 1.98–4.72). Maternal education level and household income did not significantly predict weight status (*p* ≥ 0.05). Those ahead in their maturity status are two times more likely to be overweight/obese (OR = 6.75, 95%CI = 4.38–10.41). Finally those classified in class 2 (insufficiently active, better diet quality) were less likely to be overweight/obese (OR = 0.60, 95%CI = 0.43–0.84).

**Table 2:** Configurations of behavioural risks, their observed (fo) and expected frequencies (fe), χ^2^ statistic and *p*-values.

No. of Risks	MVPA < 60 min	Fruits/Vegs All Days	Sleep < 10 h	Screen ≥ 120 min	Sugar Drinks ≥ 2 days/week	fo	fe	χ^2^	*p*-Value
0	0	0	0	0	0	1	21.44	19.48	<0.001
1	0	0	0	0	1	2	21.44	17.62	<0.001
1	0	0	0	1	0	2	21.44	17.62	<0.001
1	0	0	1	0	0	19	21.44	0.28	0.598
1	0	1	0	0	0	1	21.44	19.48	<0.001
1	1	0	0	0	0	2	21.44	17.62	<0.001
2	0	0	0	1	1	2	21.44	17.62	<0.001
2	0	0	1	0	1	11	21.44	5.08	0.024
2	0	0	1	1	0	18	21.44	0.55	0.457
2	0	1	0	0	1	3	21.44	15.86	<0.001
2	0	1	0	1	0	3	21.44	15.86	<0.001
2	0	1	1	0	0	38	21.44	12.80	<0.001
2	1	0	0	0	1	0	21.44	21.48	<0.001
2	1	0	0	1	0	3	21.44	15.86	<0.001
2	1	0	1	0	0	43	21.44	21.69	<0.001
2	1	1	0	0	0	5	21.44	12.60	<0.001
3	0	0	1	1	1	14	21.44	2.58	0.108
3	0	1	0	1	1	3	21.44	15.86	<0.001
3	0	1	1	0	1	25	21.44	0.59	0.442
3	0	1	1	1	0	52	21.44	43.57	<0.001
3	1	0	0	1	1	3	21.44	15.86	<0.001
3	1	0	1	0	1	4	21.44	14.18	<0.001
3	1	0	1	1	0	56	21.44	55.72	<0.001
3	1	1	0	0	1	1	21.44	19.49	<0.001
3	1	1	0	1	0	9	21.44	7.22	0.007
3	1	1	1	0	0	72	21.44	119.26	<0.001
4	0	1	1	1	1	56	21.44	55.72	<0.001
4	1	0	1	1	1	17	21.44	0.92	0.338
4	1	1	0	1	1	10	21.44	6.10	0.014
4	1	1	1	0	1	24	21.44	0.31	0.580
4	1	1	1	1	0	120	21.44	453.16	<0.001
5	1	1	1	1	1	67	21.44	96.84	<0.001

0 = no; 1 = yes; Example: 00000 = 0 risk behaviour; 11111 = 5 risk behaviours.

**Table 3:** Criteria used to identify the best number of latent classes.

Fit measures	Number of Classes		
	2	3	4
Pearson χ^2^	18.208	8.997	4.064
LR χ^2^	18.839	9.740	4.270
# of parameters	11	17	23
AIC	3851.165	3854.065	3860.596
BIC	3901.004	3931.090	3964.806
LMR LRT	42.152	8.873	5.333
	1 class *vs.* 2 classes	2 classes *vs.* 3 classes	3 classes *vs.* 4 classes
BLRT probability	<0.001	0.280	0.614

LR, Likelihood ratio test; AIC, Akaike information criteria; BIC, Bayesian AIC; LMR LRT, Lo-Mendell-Rubin likelihood ratio test; BLRT, Bootstrap LRT *p*-value.

**Table 4:** Behavioural, biological and demographic characteristics of the Portuguese 2-latent classes.

	Class 1 ( *n* = 242) Sedentary, Poorer Diet Quality	Class 2 ( *n* = 444) Insufficiently Active, Better Diet Quality		*p*-Value
	*n* (%)	*n* (%)		
MVPA	**	**	<0.001	
≥60 min·day^−1^	116 (47.9%)	134 (30.2%)		
<60 min·day^−1^	126 (52.1%)	310 (69.8%)		
Fruits/Vegetables			0.004	
All days	53 (21.9%)	144 (32.4%)		
<7 days	189 (78.1%)	300 (67.6%)		
Sleep time			0.051	
≥10 h·day^−1^	24 (9.9%)	26 (5.9%)		
<10 h·day^−1^	218 (90.1%)	418 (94.1%)		
Screen time			0.002	
<120 min·day^−1^	70 (28.9%)	181 (40.8%)		
≥120 min·day^−1^	172 (71.1%)	263 (59.2%)		
Sugary drinks			<0.001	
<2 days/week	0 (0.0%)	444 (100.0%)		
≥2 days/week	242 (100.0%)	0 (0.0%)		
Gender			0.005	
Girls	117 (48.4%)	264 (59.5%)		
Boys	125 (51.7%)	180 (40.5%)		
Weight status			0.059	
Normal Weight	143 (59.1%)	229 (51.6%)		
Overweight/obese	99 (40.91%)	215 (48.4%)		
Maternal education			<0.001	
<Grade 12	120 (49.6%)	197 (44.4%)		
Grade 12/diploma/technical qualification	79 (32.6%)	112 (25.2%)		
University	20 (8.7%)	88 (19.8%)		
Did not report	23 (9.5%)	47 (10.6%)		
Household income			0.106	
<12.000 €	94 (38.8%)	176 (39.6%)		
12.000 €–29.999 €	77 (31.8%)	118 (26.6%)		
≥30.000 €	18 (7.4%)	58 (13.1%)		
Did not report	53 (21.9%)	92 (20.7%)		
Maturity offset	Mean ± SD	Mean ± SD	0.292	
	−2.02 ± 0.95	−1.83 ± 0.85		

**Table 5:** Associations (coefficients, standard errors ‡, OR and 95%CI) between biological, socio-demographic characteristics, latent classes and BMI classes.

Variables	Coefficients(SE)	Odds Ratio	95%CI	*p*-Value
Sex (Male)	1.12(0.22)	3.06	1.98–4.72	<0.001
Maternal Education				
<12 Grade		Reference		
Grade12/diploma/technical	0.14(0.26)	1.15	0.70–1.90	0.550
University	0.49(0.35)	1.632	0.83–3.21	0.156
Household income				
<12.000€		Reference		
12.000 €–29.999 €	−0.25(0.26)	0.78	0.47–1.30	0.339
≥30.000 €	−0.50(0.32)	0.61	0.32–1.14	0.123
Maturity offset	1.91(0.22)	6.75	4.38–10.41	<0.001
Latent Classes (iahdq)	−0.51(0.170)	0.60	0.43–0.84	<0.001

‡, standard-errors adjusted for school clustering; iabdq, insufficiently active, better diet quality.

## 4. Discussion

This study identified lifestyle behavior profiles in Portuguese children aged 10 years based on five health behaviours (physical activity, sleep time, screen time, fruit/vegetable consumption and sugary drink consumption). The study was also designed to estimate the effects of individual and socio-demographic characteristics in predicting risk class membership.

Descriptive Configural clustering of unhealthy lifestyle behaviours indicated that a large number of children showed multiple unhealthy lifestyle behaviours but with distinct frequencies: 10% had all unhealthy lifestyle behaviours present, 78% had three or more risks, and only 0.2% did not have any unhealthy lifestyle behaviours. Low sleep time was the most prevalent of the unhealthy lifestyle behaviours observed, followed by lack of consumption of fruits and vegetables <7 days/week, physical inactivity, high screen time and consumption of sugary drinks. Sanchez *et al.* (2007), in a study of time spent in PA and watching TV, daily intake of calories from fat, and daily serving of fruits and vegetables, reported that nearly 80% of U.S. adolescents had multiple unhealthy lifestyle behaviours and almost half had at least three unhealthy lifestyle behaviours; only 2% met all guidelines [26]. Similarly, Hardy *et al.* (2012), studied five potentially obesogenic behavioral risk factors (low PA, high screen time, low fruit and vegetable intake, high soft drink consumption and high snack intake) in Australian children, and found that 51% of the boys and 43% of the girls reported three or more unhealthy lifestyle behaviours [27]. Neither of these two studies included sleep time as a health behaviour. A recent study conducted with U.S. children aged 9–12 years, and using accelerometer data (7 consecutive days) to estimate sleep time, concluded that 97% of children slept less than 10 h/day which compares well with our estimates [28]. In the same way Galland *et al.* (2012) in a review about sleep patterns in infant and children (0–12 years old) concluded that sleep duration decreases with age and the mean sleep duration to *ca.*10 years is 9 h [29]. This may explain the high number of children who did not meet the sleep recommendations.

Two consistent and significant latent classes of unhealthy lifestyle behaviours were identified. The labelling of latent classes reflects the probabilities of individuals that meet the recommendations of healthy behaviours: class 1 “sedentary, poorer diet quality”; class 2 “insufficiently active, better diet quality”. These results suggest that several healthy lifestyle factors, which can be related to the prevalence of some chronic diseases and obesity, may not occur simultaneously in all child populations. Sabbe *et al.* (2008) identified clusters of healthy behaviours based only on PA and eating habits, and found five clusters in children aged 10 years with a similar distribution of children among each cluster. However, clusters 1 (sporty healthy eaters) and 2 (sporty mixed eaters) contained more males and cluster 5 (sedentary healthy eaters) contained more females, meaning that boys tend to be more active/sporty than girls [30]. Ottevaere *et al.* (2011) also examined the prevalence and clustering of PA, sedentary behavior and dietary patterns among European adolescents, and also identified five clusters. The two clusters with the highest prevalence (49%) were similar to the two classes found in the present study (the “active, low diet quality cluster” and the “inactive, high diet quality cluster”) [31]. However, these reports used a different statistical approach (cluster analysis) than the present study (latent class analysis), making it difficult to make comparisons. Based on latent class analysis Huh *et al.* (2011) identified distinct subtypes of children with respect to their eating, physical activity patterns and weight perceptions. The final solution originated a five-class model. The majority of the children (32.1%) were most likely to be members of class “low healthy and snack food, inactive, not weight conscious”, characterized by low probability of being active, low probability of weight consciousness [32]. We were able to find only one study that focused on the same healthy behaviours in children as in the present study using the same statistical approach [12]. In that study, three latent classes (behavioral profiles) were identified and labelled as healthy (27.7%), sedentary (24.8%) and short sleepers/unhealthy eaters (47.5%). In our study, the more relevant difference between the two classes is the fact that more active children tended to drink more sugary drinks and eat less fruit and vegetables than less active children. In addition, the more active children spent more time watching TV or playing on the computer. These results may indicate that there is no association between time spent in MVPA and screen time. This result may partly explain the lower consumption of fruits and vegetables and the higher consumption of sugary drinks among most active children, since a relationship seems to exist between TV viewing and unhealthy dietary behaviours in children and adolescents [33]. Regarding sleep, a study by Magee *et al.* (2013) sampled six to seven year-old children, while the present one has 9–11 year olds and the difference found may be explained by the higher variability in sleep duration in children below 10 years old [29].

The results of our study suggest that boys and those that mature early are more likely to be overweight/obese. In Portuguese children, sex differences in obesity are usually observed [34]. A possible explanation for this difference may be due to cultural perceptions that often lead boys to engage more in MVPA. Moreover, girls are more concerned about their body image, with a desire to be lean (but not necessary fit) [35]. Girls tend to increase their awareness about their diet (to control or lose weight) [36] which, in turn, may explain their greater consumption of healthy food (such as fruits and vegetables) and lower consumption of unhealthy foods (such as sugary drinks and fast/junk foods) and less likely to be overweight/obese. On the other hand, boys tend to meet the recommendations for less screen time more than girls, which has been reported by previous investigators [37]. A possible explanation for these results may be that screen time is probably not representative of the entire spectrum of children’s sedentary behavior, and girls may choose to engage in other types of behaviours that were not assessed. The association between biological maturity status and body fat has been well reported [38,39,40,41].

The present study also indicated that children classified in class 2 (insufficiently active, better diet quality) were less likely to be overweight/obesity than those with classified in class 1 (sedentary, poorer diet quality). Regarding weight status, there is no consensus about the relationship between overweight/obesity with PA levels, as some reports show a significant and positive relationship between these variables [42], while others fail to observe any significant relationship [34]. Another possible explanation for these results may be that high quality diet may be more important to weight maintenance than physical activity or sedentary behaviours within the context of the present analysis [43].

In the present study maternal education and household income do not predict overweight and obese. The results from other studies are not always clear about the magnitude and direction of this association [44], but there is some evidence that children from higher SES tend to spend more time in sedentary activities [45], thus decreasing time spent in MVPA. With regard to food habits, children from higher SES are typically exposed to a healthier environment, with easier access to healthy food [46].

A high prevalence of overweight and obesity (≈46%) was found among Portuguese children in this study which is not in complete agreement with previous studies. For example, Vasques *et al.* (2012) found a prevalence of about 30% in children and adolescents aged 6 to 13 years using the IOTF cut points [47]. Sardinha *et al.* (2011) studied Portuguese youth aged 10 to 18 years, and reported the prevalence of overweight and obesity contrasting two cut points 22.7% and 31.7% using the IOTF and WHO references, respectively. However, when only 10 year-old children were considered, the prevalence was 45.3% for girls and 50.0% of boys using WHO cut points [48]. That report is in agreement with our results from the present study and, unfortunately reflects the trends observed in pediatric overweight and obesity, especially in Western and developed societies [49]. The high prevalence of obesity found in the present study may well be a consequence of changes in children’s lifestyles mostly linked to their nutrition and other unhealthy behaviours, namely time spent in sedentary activities [50,51,52].

This study has several limitations. Firstly, the cross-sectional design does not allow cause-effect interpretation. Secondly, latent class analysis (LCA) is a person-centered approach and the present sample comes from only one Portuguese region (North of Portugal), hence the results do not represent all Portuguese children. However, when comparing the present study sample population with information available from Portuguese children of the same age and sex, no differences were found in regard to the prevalence of overweight/obesity, the percentage of children attaining MVPA guidelines, and SES distribution [45]. Thirdly, the use of an indirect method to determine nutritional habits and screen time can be prone to error; however, these methods have been found to be reliable and valid [18,44,45] and have been used in children. Finally, this study found only two classes/profiles in Portuguese children’s behaviours, despite the fact that other studies found different solutions albeit the data analysis is somewhat different. All these results are not based in any formal behaviour theory linking physical activity, diet, screen time and sleep habits and as such are to be considered as exploratory and by no means should be generalized to any population.

Notwithstanding these limitations, the study has several important strengths: (1) the use of an objective method to estimate MVPA and sleep time; (2) the large sample size that provides detailed information about a particular age group; (3) the use of sophisticated statistical procedures to analyze configurations of behavioral risks and to identify latent classes; (4) the use of standardized methods of data collection within a robust quality control program [13]; and (5) the important contribution of its data given the lack of research on the aggregation of health behaviors in Portuguese children.

## 5. Conclusions

In summary, two classes of unhealthy lifestyle behaviours were identified, the most prevalent of which (about 65%) characterized by lower levels of PA but with healthier dietary patterns. Boys, and more mature children were more likely to be overweight/obese, but those belonging to class 2 were less likely to be overweight/obese. Children have distinct profiles of unhealthy lifestyle behaviours influenced by sex, weight status and SES. These profiles can be used to stratify children into appropriate sub-groups. Specifically, during intervention development, the sub-groups identified here could be used to improve/enhance health promotion interventions. For example, Class 1 may not be a high-priority because this sub-group had the smallest proportion of children (about 35%) who needed to reduce consumption of sugary drinks and time spent in watching TV or playing video games. Class 2 needs to increase physical activity. A message to “eat fruits and vegetables” should be a common theme relevant to all the sub-groups, since the consumption of fruits and vegetables was low for both. When planning intervention strategies, designing and adapting specific communication messages to increase fruits and vegetables, decrease sedentary activity, and increase sleep time should set the foundation for improved health behaviours. Although inconsistencies exist between behavioural issues and BMI, future research efforts perhaps can tease or tweak these issues and provide more clarity to potential associations. Overweight and obesity are complex issues, undoubtedly requiring more intense research to delineate cause and effect on an individual basis. For now, only group effects (clusters) seem to be elucidating the relationship. Future research should also focus on longitudinal analysis to study change and stability of obesogenic behaviors profiles across childhood and adolescence, as well as possible mediating roles of familial influences, peer-pressure, school level contexts as well as built environmental correlates.

## References

[1] Carson V., Stone M., Faulkner G. (2014). Patterns of Sedentary Behavior and Weight Status among Children. Pediatr. Exerc. Sci..

[2] Kell K.P., Cardel M.I., Bohan Brown M.M., Fernández J.R. (2014). Added sugars in the diet are positively associated with diastolic blood pressure and triglycerides in children. Am. J. Clin. Nutr..

[3] Leech R.M., McNaughton S.A., Timperio A. (2014). The clustering of diet, physical activity and sedentary behavior in children and adolescents: A review. Int. J. Behav. Nutr. Phys. Act..

[4] Chen X., Beydoun M.A., Wang Y. (2008). Is sleep duration associated with childhood obesity? A systematic review and meta-analysis. Obesity.

[5] Knutson K.L. (2010). Sleep duration and cardiometabolic risk: A review of the epidemiologic evidence. Best Pract. Res. Clin. Endocrinol. Metab..

[6] Xi B., He D., Zhang M., Xue J., Zhou D. (2014). Clinical review: Short sleep duration predicts risk of metabolic syndrome: A systematic review and meta-analysis. Sleep Med. Rev..

[7] Stamatakis E., Coombs N., Jago R., Gama A., Mourão I., Nogueira H., Rosado V., Padez C. (2013). Associations between indicators of screen time and adiposity indices in Portuguese children. Prev. Med..

[8] Valente H., Teixeira V., Padrão P., Bessa M., Cordeiro T., Moreira A., Mitchell V., Lopes C., Mota J., Moreira P. (2011). Sugar-sweetened beverage intake and overweight in children from a Medierranean country. Public Health Nutr..

[9] Gomes T.N., Katzmarzyk P.T., dos Santos F.K., Souza M., Pereira S., Maia J.A. (2014). Overweight and obesity in Portuguese children: Prevalence and correlates. Int. J. Environ. Res. Public Health.

[10] Fernández-Alvira J.M., de Bourdeaudhuij I., Singh A.S., Vik F.N., Manios Y., Kovacs E., Jan N., Brug J., Moreno L.A. (2013). Clustering of energy balance-related behaviors and parental education in European children: The ENERGY-project. Int. J. Behav. Nutr. Phys. Act..

[11] Jago R., Fox K.R., Page A.S., Brockman R., Thompson J.L. (2010). Physical activity and sedentary behaviour typologies of 10–11 year olds. Int. J. Behav. Nutr. Phys. Act..

[12] Magee C.A., Caputi P., Iverson D.C. (2013). Patterns of health behaviours predict obesity in Australian children. J. Paediatr. Child. Health.

[13] Katzmarzyk P.T., Barreira T.V., Broyles S.T., Champagne C.M., Chaput J.P., Fogelholm M., Hu G., Johnson W.D., Kuriyan R., Kurpad A. (2013). The International Study of Childhood Obesity, Lifestyle and the Environment (ISCOLE): Design and methods. BMC Public Health.

[14] World Health Organization (1995). Physical Status: The Use and Interpretation of Anthropometry: Report of a WHO Expert Committee.

[15] Mirwald R.L., Baxter-Jones A.D.G., Bailey D.A., Beunen G.P. (2002). An assessment of maturity from anthropometric measurements. Med. Sci. Sports Exerc..

[16] Evenson K.R., Catellier D.J., Gill K., Ondrak K.S., McMurray R.G. (2008). Calibration of two objective measures of physical activity for children. J. Sports Sci..

[17] World Health Organization (2010). Global Recommendations on Physical Activity for Health.

[18] Tudor-Locke C., Barreira T.V., Schuna J.M., Mire E.F., Katzmarzyk P.T. (2014). Fully automated waist-worn accelerometer algorithm for detecting children’s sleep-period time separate from 24-h physical activity or sedentary behaviors. Appl. Physiol. Nutr. Metab..

[19] Barreira T.V., Schuna J.M., Mire E.F., Katzmarzyk P.T., Chaput J.P., Leduc G., Tudor-Locke C. (2015). Identifying children’s nocturnal sleep using 24-h waist accelerometry. Med. Sci. Sports Exerc..

[20] National Sleep Foundation. Children and Sleep.

[21] Currie C., Gabhainn S.N., Godeau E., Roberts C., Smith R., Currie D., Pickett W., Richter M., Morgan A., Barnekow V. (2008). Inequalities in Young People’s Health: HBSC International Report from the 2005/06 Survey. Health Policy for Children and Adolescents, No. 5.

[22] Barlow S.E. (2007). Expert committee recommendations regarding the prevention, assessment, and treatment of child and adolescent overweight and obesity: Summary report. Pediatrics.

[23] Schrepp M. (2006). The use of configural frequency analysis for explorative data analysis. Br. J. Math. Stat. Psychol..

[24] Geiser C. (2013). Data Analysis with Mplus.

[25] Wang J., Wang X. (2012). Structural Equation Modelling: Applications Using Mplus.

[26] Sanchez A., Norman G.J., Sallis J.F., Calfas K.J., Cella J., Patrick K. (2007). Patterns and correlates of physical activity and nutrition behaviors in adolescents. Am. J. Prev. Med..

[27] Hardy L.L., Grunseit A., Khambalia A., Bell C., Wolfenden L., Milat A.J. (2012). Co-occurrence of obesogenic risk factors among adolescents. J. Adolesc. Health.

[28] Wong W.W., Ortiz C.L., Lathan D., Moore L.A., Konzelmann K.L., Adolph A.L., Simth E.O., Butte N.F. (2013). Sleep duration of underserved minority children in a cross-sectional study. BMC Public Health.

[29] Galland B.C., Taylor B.J., Elder D.E., Herbison P. (2012). Clinical Review: Normal sleep patterns in infants and children: A systematic review of observational studies. Sleep Med. Rev..

[30] Sabbe D., de Bourdeaudhuij I., Legiest E., Maes L. (2008). A cluster-analytical approach towards physical activity and eating habits among 10-year-old children. Health Educ. Res..

[31] Ottevaere C., Huybrechts I., Benser J., de Bourdeaudhuij I., Cuenca-Garcia M., Dallongeville J., Zaccaria M., Gottrand F., Kersting M., Rey-López J.P. (2011). Clustering patterns of physical activity, sedentary and dietary behavior among European adolescents: The HELENA study. BMC Public Health.

[32] Huh J., Riggs N.R., Spruijt-Metz D., Chou C., Huang Z., Pentz M. (2011). Identifying Patterns of Eating and Physical Activity in Clildren: A Latent Class Analysis of Obesity Risk. Obesity.

[33] Pearson N., Biddle S.J.H. (2011). Sedentary Behavior and Dietary Intake in Children, Adolescents, and Adults: A Systematic Review. Am. J. Prev. Med..

[34] Martins D., Maia J., Seabra A., Garganta R., Lopes V., Katzmarzyk P., Beunen G. (2010). Correlates of changes in BMI of children from the Azores islands. Int. J. Obes. (Lond.).

[35] Kanaan M.N., Afifi R.A. (2010). Gender differences in determinants of weight-control behaviours among adolescents in Beirut. Public Health Nutr..

[36] Harter S., Damon W., Lerner R.M. (2006). The Self. Handbook of Child Psychology.

[37] Jago R., Thompson J.L., Sebire S.J., Wood L., Pool L., Zahra J., Lawlor D.A. (2014). Cross-sectional associations between the screen-time of parents and young children: Differences by parent and child gender and day of the week. Int. J. Behav. Nutr. Phys. Act..

[38] Coelho-E-Silva M.J., Vaz E.R., Cyrino E.S., Fernandes R.A., Valente-Dos-Santos J., Machado-Rodrigues A., Malina R.M. (2013). Nutritional status, biological maturation and cardiorespiratory fitness in Azorean youth aged 11–15 years. BMC Public Health.

[39] Adair L.S., Gordon-Larsen P. (2001). Maturational timing and overweight prevalence in US adolescent girls. Am. J. Public Health.

[40] Beunen G.P., Malina R.M., Lefevre J.A., Claessens A.L., Renson R., Vanreusel B. (1994). Adiposity and biological maturity in girls 6–16 years of age. Int. J. Obes. Relat. Metab. Disord..

[41] Santos J.R., Duarte J.P., Mota J. (2006). Association between overweight and early sexual maturation in Portuguese boys and girls. Ann. Hum. Biol..

[42] Utter J., Scragg R., Schaaf D., Fitzgerald E., Wilson N. (2007). Correlates of body mass index among a nationally representative sample of New Zealand children. Int. J. Pediatr. Obes..

[43] Gomes T.N., dos Santos F.K., Santos D., Chaves R.N., Souza M., Katzmarzyk P.T., Maia J. (2014). “Fat-But-Active”: Does Physical Activity Play a Significant Role in Metabolic Syndrome Risk among Children of Different BMI Categories?. J. Diabetes Metab..

[44] Steele R.M., van Sluijs E.M.F., Sharp S.J., Landsbaugh J.R., Ekelund U., Griffin S.J. (2010). An investigation of patterns of children’s sedentary and vigorous physical activity throughout the week. Int. J. Behav. Nutr. Phys. Act..

[45] Atkin A.J., Corder K., Ekelund U., Wijndaele K., Griffin S.J., van Sluijs E.M. (2013). Determinants of change in children’s sedentary time. PLoS ONE.

[46] Elsenburg L.K., Corpeleijn E., van Sluijs E.M.F., Atkin A.J. (2014). Clustering and Correlates of Multiple Health Behaviours in 9–10 Year Old Children. PLoS ONE.

[47] Vasques C., Mota M., Correia T., Lopes V. (2012). Prevalence of overweight/obesity and its association with sedentary behavior in children. Rev. Port. Cardiol..

[48] Sardinha L.B., Santos R., Vale S., Silva A.M., Ferreira J.P., Raimundo A.M., Moreira H., Baptista F., Mota J. (2011). Prevalence of overweight and obesity among Portuguese youth: A study in a representative sample of 10–18-year-old children and adolescents. Int. J. Pediatr. Obes..

[49] Janssen I., Katzmarzyk P.T., Boyce W.F., Vereecken C., Mulvihill C., Roberts C., Currie C., Pickett W. (2005). Comparison of overweight and obesity prevalence in school-aged youth from 34 countries and their relationships with physical activity and dietary patterns. Obes. Rev..

[50] Cecchini M., Sassi F., Lauer J.A., Lee Y.Y., Guajardo-Barron V., Chisholm D. (2010). Tackling of unhealthy diets, physical inactivity, and obesity: Health effects and cost-effectiveness. Lancet.

[51] Vereecken C.A., Maes L. (2003). A Belgian study on the reliability and relative validity of the Health Behaviour in School-Aged Children food-frequency questionnaire. Public Health Nutr..

[52] Schmitz K.H., Harnack L., Fulton J.E., Jacobs D.R., Gao S., Lytle L.A. (2004). Reliability and validity of a brief questionnaire to assess television viewing and computer use by middle school children. J. School Health.

